# Population-Based Long-term Cardiac-Specific Mortality Among Patients With Major Gastrointestinal Cancers

**DOI:** 10.1001/jamanetworkopen.2021.12049

**Published:** 2021-06-17

**Authors:** Daryl Ramai, Joseph Heaton, Michele Ghidini, Saurabh Chandan, Mohamed Barakat, Banreet Dhindsa, Amaninder Dhaliwal, Antonio Facciorusso

**Affiliations:** 1 Department of Internal Medicine, Brooklyn Hospital Center, Brooklyn, New York; 2 Division of Medical Oncology, Fondazione IRCCS Ca' Granda, Ospedale Maggiore Policlinico, Milan, Italy; 3 Division of Gastroenterology & Hepatology, CHI Health Creighton University Medical Center, Omaha, Nebraska; 4 Division of Gastroenterology, The Brooklyn Hospital Center, Brooklyn, New York; 5 Gastroenterology & Hepatology, University of Nebraska Medical Center, Omaha; 6 Division of Gastroenterology, Moffitt Cancer Center, University of South Florida, Tampa; 7 Section of Gastroenterology, Department of Medical Sciences, University of Foggia, Foggia, Italy

## Abstract

**Importance:**

Patients with major gastrointestinal (GI) cancers are at long-term risk for cardiac disease and mortality.

**Objective:**

To investigate the cardiac-specific mortality rate among individuals with major GI cancers and the association of radiation and chemotherapy with survival outcomes in the United States.

**Design, Setting, and Participants:**

This US cohort study included individual patient-level data of men and women older than 18 years with 5 major gastrointestinal cancers, including colorectal, esophageal, gastric, pancreatic, and hepatocellular cancer from 1990 to 2016. Data was extracted from the Surveillance, Epidemiology, and End Results (SEER) national cancer database. Data cleaning and analyses were conducted between November 2020 and March 2021.

**Exposures:**

Patients received chemotherapy, radiotherapy, or a combination of adjuvant therapy for major GI cancers.

**Main Outcomes and Measures:**

The primary outcome was cardiac-specific mortality. Examined factors associated with cardiac mortality included age, sex, race, tumor location, tumor grade, SEER stage, TNM (seventh edition) staging criteria, cancer treatment (ie, the use of radiation, chemotherapy, or surgery), survival months, and cause of death.

**Results:**

A total of 359 032 patients (mean [SD] age at baseline, 65.1 [12.9] years; 186 921 [52.1%] men) with GI cancers were analyzed, including 313 940 patients (87.4%) with colorectal cancer, 7613 patients (2.1%) with esophageal cancer, 21 048 patients (5.9%) with gastric cancer, 7227 patients (2.0%) with pancreatic cancer, and 9204 patients (2.6%) with hepatocellular cancer. Most cancers were localized except pancreatic cancer, which presented with regional and distant involvement (3680 cancers [50.9%]). Overall, all major gastrointestinal tumors were associated with increased risk of cardiac mortality compared with noncardiac mortality (median survival time: 121 [95% CI, 120-122] months vs 287 [95% CI, 284.44-290] months). Patients with hepatocellular cancer had the lowest cardiac-specific median survival time (98 [95% CI, 90-106] months), followed by pancreatic cancer (105 [95% CI, 98-112] months), esophageal cancer (113 [95% CI, 107-119] months), gastric cancer (113 [95% CI, 110-116] months), and colorectal cancer (122 [95% CI, 121-123] months). At 15 years of follow up, the use of only chemotherapy, only radiation, or radiation and chemotherapy combined was associated with poor survival rates from cardiac causes of death (eg, colorectal: chemotherapy, 0 patients; radiation, 1 patient [1.9%]; radiation and chemotherapy, 3 patients [2.7%]).

**Conclusions and Relevance:**

These findings suggest that among patients with major gastrointestinal cancers, cardiac disease is a significant cause of mortality. The use of only chemotherapy, only radiation, or both was associated with higher cardiac mortality.

## Key Points

**Question:**

What is the frequency of cardiac-specific mortality among individuals with major gastrointestinal (GI) cancers?

**Findings:**

In this cohort study of 359 032 patients with GI cancer, patients with cancer had a higher risk of cardiac-specific mortality compared with other causes of mortality. Chemotherapy, radiation, or a combination of adjuvant therapy were associated with higher cardiac mortality.

**Meaning:**

This cohort study found that patients with major GI cancers were at high risk for cardiac mortality.

## Teaser

This cohort study examines the associations of major gastrointestinal cancers with cardiac-specific mortality.

## Introduction

Gastrointestinal (GI) cancers represent 26% of the global incidence of cancer and 35% of all cancer-related deaths, with approximately 4.8 million new cases and 3.4 million deaths each year.^1^ The major malignant conditions of the GI tract include stomach (approximately 1.0 million new cases in 2018), esophagus (570 000 cases in 2018), pancreas (460 000 cases in 2018), and colorectum (1.8 million cases in 2018).^1^ Based on age and population change, the worldwide number of new cases of GI cancers is expected to increase by 58% to 7.8 million cases, and deaths from GI cancers are expected to increase by 73% to 5.6 million deaths.^2^ In the past decade, there have been positive developments in the detection and control of GI cancers.^3^

The stage at presentation and tumor burden dictates treatment regimens. Although endoscopic treatment is possible in early cases, surgery is more commonly used in patients with advanced stages of cancer or tumor burden.^3^ For example, most patients with pancreatic cancer present with advanced tumor stage, yet only a small portion of patients undergo curative surgery.^4,5^ Similarly, more than half of patients with colorectal cancer present with regional or distant metastasis, compelling adjuvant radiation and chemotherapy.^6^

Mortality from GI cancers may occur from primary cancer-specific causes, chronic obstructive pulmonary disorder, cardiovascular disease, secondary cancers, and sequelae of chemotherapeutic agents. Overall and cause-specific mortalities have been associated with diabetes, coffee consumption, smoking, and grip strength.^7,8,9,10^ However, the risks of cardiac mortality associated with GI cancers are not well defined. To this end, we aim to determine the rate of cardiac-specific mortality in patients with esophageal, gastric, pancreatic, hepatocellular, and colorectal cancer (CRC) in the United States.

## Methods

This cohort study used data from the Surveillance, Epidemiology and End Results Registry (SEER). SEER data are publicly available, deidentified, and exempt from institutional review board approval and informed consent, per SEER guidelines. This report follows the Strengthening the Reporting of Observational Studies in Epidemiology (STROBE) reporting guideline for observational studies.

We defined cardiac-specific mortality as an overarching umbrella term to include various cardiac diseases that led to death. Data regarding patients diagnosed with esophageal, gastric, pancreatic, hepatocellular, and CRC between 1990 and 2016 were extracted from SEER. Survival data were also determined within this study period. Data were collected from 18 US registries, which, in aggregate, represent nearly 28% of the US population.^11^

The demographic variables of interest were patient sex, age at diagnosis, race, and year of diagnosis. We examined disease burden at presentation associated with race. Race was recorded as White, African American, American Indian or Alaska Native, and Asian or Pacific Islander. The age of patients was treated as categorical variables. Pediatric patients (age <18 years) were excluded from this study, since diagnostic and therapeutic interventions may differ compared with adults.

Clinical variables included disease-specific survival and treatments. Therapeutic interventions were divided into radiation, chemotherapy, surgery, and combined regimens. Pathological information included tumor grade and lymph node involvement using the American Joint Committee on Cancer staging criteria (seventh edition) and SEER summary staging.

Data cleaning and analyses were conducted between November 2020 and March 2021. A complete-case method was used for missing data. Reviewing the data showed no evidence of any patterns that might indicate a systematic association between the missing data and any independent or dependent variables in the study. Additionally, we used a Little test of the expectation-maximization estimated statistics to determine the randomness of missing values, and we assumed that missing data were completely random was reasonable and that the possible effects of selection bias were minimal.

### Statistical Analysis

SEER*Stat statistical software version 8.3.6 (National Cancer Institute) was used to perform case listing and data extraction. SPSS Statistics version 25 (IBM) was used for analysis. Kaplan-Meier estimations were performed to compare cardiac-specific and noncardiac-specific survival rates through log-rank tests. For each cancer type, Cox proportion hazard multivariable regression modeling was used to determine factors associated with mortality. The dependent variable was cardiac mortality, while independent variables used for regression analysis included sex, age (continuous variable), race, tumor grade, SEER summary stage, T stage, N stage, chemotherapy alone, radiation alone, surgery alone, combined chemotherapy and radiation, combined chemotherapy and surgery, combined radiation and surgery, and combined chemotherapy, radiation, and surgery.

Cox proportion hazard assumptions were evaluated by examining Schoenfeld residuals. Cox proportion hazard models were deemed valid if the hazard was reasonably constant over time. We excluded patients with unknown survival duration. Hypothesis tests were 2-sided, and statistical significance was set at *P* < .05.

## Results

### Patient Characteristics

A total of 359 032 patients (mean [SD] age at baseline, 65.1 [12.9] years; 186 921 [52.4%] men) with major GI cancer were included in the SEER from 1990 to 2016, including 313 940 patients (87.4%) with colorectal cancer, 7613 patients (2.1%) with esophageal cancer, 21 048 patients (5.9%) with gastric cancer, 7227 patients (2.0%) with pancreatic cancer, and 9204 patients (2.6%) with hepatocellular cancer.

Most tumors occurred among White patients (CRC: 255 017 patients [81.2%]; esophageal cancer: 6563 patients [86.2%]; gastric cancer: 14 012 patients [66.6%]; pancreatic cancer: 5813 patients [80.4%]; hepatocellular cancer: 5967 patients [64.8%]). Additional patient demographic information are reported in Table 1.

**Table 1.** Participant Characteristics

Variables	No. (%) (N = 359 032)				
	Colorectal (n = 313 940)	Esophageal (n = 7613)	Gastric (n = 21 048)	Pancreatic (n = 7227)	Hepatocellular (n = 9204)
**Characteristic**					
Sex					
Men	159 249 (50.7)	5773 (75.8)	11 823 (56.2)	3524 (48.8)	6552 (71.2)
Women	154 691 (49.3)	1840 (24.2)	9225 (43.8)	3703 (51.2)	2652 (28.8)
Race					
White	255 017 (81.2)	6563 (86.2)	14 012 (66.6)	5813 (80.4)	5967 (64.8)
Black	30 716 (9.8)	661 (8.7)	2797 (13.3)	771 (10.7)	814 (8.8)
Asian or Pacific Islander	24 600 (7.8)	322 (4.2)	3979 (18.9)	575 (8.0)	2284 (24.8)
American Indian or Alaska Native	1826 (0.6)	46 (0.6)	150 (0.7)	39 (0.5)	106 (1.2)
Unknown	1781 (0.6)	21 (0.3)	110 (0.5)	29 (0.4)	33 (0.4)
Age, y					
18-19	88 (<0.1)	0	16 (0.1)	18 (0.2)	20 (0.2)
20-24	441 (0.1)	4 (0.1)	35 (0.2)	45 (0.6)	46 (0.5)
25-29	1119 (0.4)	21 (0.3)	109 (0.5)	70 (1.0)	53 (0.6)
30-34	2481 (0.8)	28 (0.4)	249 (1.2)	148 (2.0)	84 (0.9)
35-39	4943 (1.6)	86 (1.1)	506 (2.4)	227 (3.1)	152 (1.7)
40-44	9681 (3.1)	162 (2.1)	858 (4.1)	384 (5.3)	331 (3.6)
45-49	16 940 (5.4)	426 (5.6)	1334 (6.3)	547 (7.6)	795 (8.6)
50-54	30 085 (9.6)	784 (10.3)	1760 (8.4)	822 (11.4)	1549 (16.8)
55-59	33 975 (10.8)	1099 (14.4)	2231 (10.6)	889 (12.3)	1813 (19.7)
60-64	38 731 (12.3)	1261 (16.6)	2575 (12.2)	948 (13.1)	1576 (17.1)
65-69	45 006 (14.3)	1335 (17.5)	3137 (14.9)	1003 (13.9)	1141 (12.4)
70-74	45 123 (14.4)	1127 (14.8)	3096 (14.7)	894 (12.4)	841 (9.1)
75-79	41 083 (13.1)	768 (10.1)	2602 (12.4)	671 (9.3)	490 (5.3)
80-84	28 100 (9.0)	378 (5.0)	1691 (8.0)	380 (5.3)	234 (2.5)
>85%	16 144 (5.1)	134 (1.8)	849 (4.0)	181 (2.5)	79 (0.9)
Tumor grade					
Differentiated					
Well I	34 897 (11.1)	661 (8.7)	2020 (9.6)	1622 (22.4)	1911 (20.8)
Moderately II	194 869 (62.1)	2768 (36.4)	4970 (23.6)	1654 (22.9)	2296 (24.9)
Poorly III	38 791 (12.4)	2310 (30.3)	7697 (36.6)	750 (10.4)	660 (7.2)
Undifferentiated, IV	2619 (0.8)	136 (1.8)	425 (2.0)	82 (1.1)	81 (0.9)
Unknown	42 764 (13.6)	1738 (22.8)	5936 (28.2)	3119 (43.2)	4256 (46.2)
SEER summary stage					
Localized	139 433 (44.4)	3198 (42.0)	10 037 (47.7)	2073 (28.7)	6637 (72.1)
Regional	97 571 (31.1)	2302 (30.2)	5234 (24.9)	2609 (36.1)	1340 (14.6)
Distant	9440 (3.0)	471 (6.2)	826 (3.9)	1071 (14.8)	177 (1.9)
Unknown	139 433 (100)	582 (7.6)	1291 (6.1)	601 (8.3)	559 (6.1)
TNM stage (T)					
T1	8451 (2.7)	517 (6.8)	1066 (5.1)	278 (3.8)	1218 (13.2)
T2	5743 (1.8)	159 (2.1)	556 (2.6)	367 (5.1)	541 (5.9)
T3	16 031 (5.1)	377 (5.0)	766 (3.6)	569 (7.9)	118 (1.3)
T4	51 (<0.1)	0	107 (0.5)	69 (1.0)	13 (0.1)
Unknown	283 664 (90.4)	6560 (86.2)	18 553 (88.1)	5944 (82.2)	7314 (79.5)
TNM stage (N)					
N0	25 352 (8.1)	714 (9.4)	2224 (10.6)	908 (12.6)	1858 (20.2)
≥N1	313 (0.1)	400 (5.3)	647 (3.1)	433 (6.0)	140 (1.5)
Unknown	288 275 (91.8)	6499 (85.4)	18 177 (86.4)	5886 (81.4)	7206 (78.3)
**Cancer treatment**					
Chemotherapy only					
Received	704 (0.2)	132 (1.7)	384 (1.8)	448 (6.2)	1576 (17.1)
Did not receive	313 236 (99.8)	7481 (98.3)	20 664 (98.2)	6779 (93.8)	7628 (82.9)
Radiation only					
Received	295 (0.1)	176 (2.3)	48 (0.2)	54 (0.7)	93 (1.0)
Did not receive	313 645 (99.9)	7437 (97.7)	21 000 (99.8)	7173 (99.3)	9111 (99.0)
Surgery only					
Received	209 788 (66.8)	2443 (32.1)	12 603 (59.9)	2985 (41.3)	4379 (47.6)
Did not receive	104 152 (33.2)	5170 (67.9)	8445 (40.1)	4242 (58.7)	4825 (52.4)
Combined radiation and chemotherapy					
Received	2263 (0.7)	1886 (24.8)	364 (1.7)	210 (2.9)	72 (0.8)
Did not receive	311677 (99.3)	5727 (75.2)	20 684 (98.3)	7017 (97.1)	9132 (99.2)
Combined radiation and surgery					
Received	3608 (1.1)	97 (1.3)	339 (1.6)	119 (1.6)	61 (0.7)
Did not receive	310 332 (98.9)	7516 (98.7)	20 709 (98.4)	7108 (98.4)	9143 (99.3)
Combined chemotherapy and surgery					
Received	57705 (18.4)	262 (3.4)	1995 (9.5)	722 (10.0)	1706 (18.5)
Did not receive	256235 (81.6)	7351 (96.6)	19 053 (90.5)	6505 (90.0)	7498 (81.5)
Combined chemotherapy, radiation, and surgery					
Received	31 621 (10.1)	1988 (26.1)	3294 (15.6)	1178 (16.3)	40 (0.4)
Did not receive	282 319 (89.9)	5625 (73.9)	17 754 (84.4)	6049 (83.7)	9164 (99.6)
No therapy					
Received	7956 (2.5)	629 (8.3)	2021 (9.6)	1511 (20.9)	1277 (13.9)
Did not receive	305984 (97.5)	6984 (91.7)	19027 (90.4)	5716 (79.1)	7927 (86.1)

Abbreviation: SEER, Surveillance, Epidemiology, and End Results.

### Tumor Characteristics

A total of 139 433 patients with CRC (44.4%) presented with localized cancer, while 3198 patients with esophageal cancer (42.0%) presented with localized cancer, 10 037 patients with gastric cancer (47.7%) presented with localized cancer, and 6637 patients with hepatocellular cancer (72.1%) presented with localized cancer. Most patients with pancreatic tumors (3680 patients [50.9%]) presented with regional or distant cancer. Lymph node involvement was observed in 313 patients with CRC (0.1%), 400 patients with esophageal cancer (5.3%), 647 patients with gastric cancer (3.1%), 433 patients with pancreatic cancer (6.0%), and 140 patients with hepatocellular cancer (1.5%).

### Overall Survival

Among all GI tumors, cardiac-specific disease had significantly higher mortality than other causes of mortality (median survival time: 121 [95% CI, 120-122] months vs 287 [95% CI, 284-290] months). Among patients with CRC, cardiac-specific median survival was 122 (95% CI, 121-123) months vs other-cause mortality of 287 (95% CI, 284-290) months (*P* < .001). The cardiac-specific survival rate among patients with CRC was 25 881 patients (89.3%) at 10 years and 1428 patients (4.9%) at 15 years, compared with other-cause mortality of 278 258 patients (97.7%) at 10 years and 177 543 patients (62.3%) at 15 years. Patients with esophageal cancer had a cardiac-specific median survival of 113 (95% CI, 107-119) months vs other-cause mortality of 271 (95% CI, 251-291) months (*P* < .001). The cardiac-specific survival rate among patients with esophageal cancer was 529 patients (85.7%) at 10 years and 24 patients (3.9%) at 15 years, compared with other-cause mortality of 6793 patients (97.1%) at 10 years and 4252 patients (60.8%) at 15 years. Patients with gastric cancer had a cardiac-specific median survival of 113 (95% CI, 110-116) months vs other-cause mortality of 278 (95% CI, 269-287) months (*P* < .001). Among patients with gastric cancer, the cardiac-specific survival rate was 1420 patients (87.1%) at 10 years and 60 patients (3.7%) at 15 years, compared with other-cause mortality of 18 917 patients (97.5%) at 10 years and 12 030 patients (62.0%) at 15 years.

Patients with pancreatic cancer had the lowest cardiac-specific median survival, at 105 (95% CI, 98-112) months vs other-cause mortality of 293 (95% CI, 271-315) months (*P* < .001). Patients with pancreatic cancer had a cardiac-specific survival rate of 261 patients (84.6%) at 10 years and 9 patients (2.8%) at 15 years compared with other-cause mortality of 6793 patients (98.2%) at 10 years and 4712 patients (68.1%) at 15 years. Patients with hepatocellular carcinoma a cardiac-specific median survival of 98 (95% CI, 90-106) months. We were unable to determine median survival for other-cause mortality, because most of those patients did not experience mortality. The cardiac-specific survival rate among these patients was 178 patients (77.1%) at 10 years and 6 patients (2.6%) at 15 years compared with other-cause mortality of 8751 patients (97.5%) at 10 years and 6659 patients (74.2%) at 15 years (Table 2).

**Table 2.** Median Survival Time According to Cancer Type

Cancer	Median survival (95% CI), mo		*P* value
	Cardiac COD	Noncardiac COD	
Colorectal	122 (121-123)	287 (284-290)	<.001
Esophageal	113 (107-119)	271 (251-291)	<.001
Gastric	113 (110-116)	278 (269-287)	<.001
Pancreatic	105 (98-112)	293 (271-315)	<.001
Hepatocellular	98 (90-106)	NA	<.001

Abbreviations: COD, cause of death; NA, not available.

### Association of Multimodal Therapy With Survival

We assessed the association of chemotherapy or radiation with cancer survival. At 10 and 15 years, patients with CRC had lower cardiac-specific mortality vs noncardiac survival after being treated with chemotherapy only (10 years: 18 patients [84.2%] vs 669 patients [98.0%]; 15 years: 0 patients vs 511 patients [74.8%]), radiation only (10 years: 28 patients [82.1%] vs 253 patients [97.5%]; 15 years: 1 patient [1.9%] vs 154 patients [59.0%]), and combined radiation and chemotherapy (10 years: 88 patients [73.6%] vs 2101 patients [98.1%]; 15 years: 3 patients [2.7%]; vs 1476 patients [68.9%]). Similar findings were observed for esophageal, gastric, pancreatic, and hepatocellular cancer (Table 3).

**Table 3.** Survival Rate at 10 and 15 Years Among Patients with Gastrointestinal Cancer by Cancer Type and Treatment Received

Treatment	Patients, No. (%)			
	Cardiac COD		Noncardiac COD	
	10-y survival	15-y survival	10-y survival	15-y survival
**Colorectal cancer**				
Overall	25 881 (89.3)	1428 (4.9)	278 528 (97.7)	177 543 (62.3)
Chemotherapy only	18 (84.2)	0	669 (98.0)	511 (74.8)
Radiation only	28 (82.1)	1 (1.9)	254 (97.5)	154 (59.0)
Surgery only	20 588 (89.0)	1194 (5.2)	181 629 (97.3)	109 479 (58.7)
Combined				
Radiation and chemotherapy	88 (73.6)	3 (2.7)	2101 (98.1)	1476 (68.9)
Radiation and surgery	355 (93.1)	14 (3.6)	3145 (97.5)	1688 (52.3)
Chemotherapy and surgery	2941 (91.5)	156 (4.9)	53 802 (98.7)	38 775 (71.2)
Chemotherapy, radiation, and surgery	1312 (89.9)	35 (2.4)	29 794 (98.8)	21 468 (71.2)
No therapy	551 (88.0)	34 (5.4)	7147 (97.5)	5093 (69.5)
**Esophageal cancer**				
Overall	529 (85.7)	24 (3.9)	6793 (97.1)	4252 (60.8)
Chemotherapy only	10 (90.5)	0	120 (99.1)	64 (52.6)
Radiation only	18 (81.8)	0	140 (90.7)	69 (45.0)
Surgery only	155 (87.0)	8 (4.6)	2219 (98.0)	1460 (64.4)
Combined				
Radiation and chemotherapy	169 (83.4)	8 (3.8)	1609 (95.6)	854 (50.8)
Radiation and surgery	6 (66.7)	0	85 (96.3)	51 (58.1)
Chemotherapy and surgery	11 (91.3)	1 (11.0)	247 (98.7)	179 (71.7)
Chemotherapy, radiation, and surgery	112 (88.7)	6 (4.7)	1814 (97.4)	1207 (64.8)
No therapy	49 (85.7)	1 (1.3)	559 (97.7)	345 (60.2)
**Gastric cancer**				
Overall	1430 (87.1)	60 (3.7)	18 917 (97.5)	12 030 (62.0)
Chemotherapy only	13 (72.2)	0	357 (97.5)	286 (78.1)
Radiation only	1 (100)	0	40 (85.7)	18 (38.7)
Surgery only	1039 (87.2)	46 (3.8)	11 098 (97.2)	6685 (58.6)
Combined				
Radiation and chemotherapy	37 (85.9)	1 (3.4)	308 (96.1)	199 (62.0)
Radiation and surgery	23 (88.2)	1 (2.6)	309 (98.7)	190 (60.8)
Chemotherapy and surgery	53 (85.2)	4 (6.1)	1901 (98.3)	1349 (69.8)
Chemotherapy, radiation, and surgery	134 (93.0)	3 (2.2)	3093 (98.2)	2272 (72.1)
No therapy	130 (83.4)	5 (3.3)	1812 (97.1)	1184 (63.5)
**Pancreatic cancer**				
Overall	261 (84.6)	9 (2.8)	6793 (98.2)	4712 (68.1)
Chemotherapy only	12 (85.2)	0	425 (97.8)	340 (78.4)
Radiation only	2 (100)	0	51 (97.8)	27 (51.0)
Surgery only	97 (81.9)	5 (3.9)	2833 (98.8)	2124 (74.1)
Combined				
Radiation and chemotherapy	10 (100)	0	195 (97.7)	120 (60.0)
Radiation and surgery	5 (100)	2 (30.0)	110 (96.2)	64 (56.1)
Chemotherapy and surgery	13 (78.6)	1 (5.9)	704 (99.7)	577 (81.7)
Chemotherapy, radiation, and surgery	22 (77.4)	0	1134 (98.6)	760 (66.1)
No therapy	102 (87.5)	2 (1.7)	1342 (96.2)	754 (54.0)
**Hepatocellular cancer**				
Overall	178 (77.1)	6 (2.6)	8751 (97.5)	6659 (74.2)
Chemotherapy only	19 (75.5)	0	1511 (97.4)	1173 (75.6)
Radiation only	2 (60.0)	0	88 (97.3)	64 (71.7)
Surgery only	92 (74.5)	3 (2.1)	4138 (97.2)	3132 (73.6)
Combined				
Radiation and chemotherapy	2 (100)	0	70 (100)	45 (64.6)
Radiation and surgery	0	0	60 (98.1)	48 (79.1)
Chemotherapy and surgery	25 (80.3)	0	1644 (98.2)	1300 (77.6)
Chemotherapy, radiation, and surgery	0	0	40 (100)	38 (95.7)
No therapy	39 (82.6)	4 (8.2)	1200 (97.6)	873 (71.0)

At 15 years, multimodal therapy with combined chemotherapy and radiation had low cardiac survival rates but higher noncardiac survival rates (esophageal: 8 patients [3.8%] vs 854 patients [50.8%]; gastric: 1 patient [3.4%] vs 199 patients [62.0%]; pancreatic: 0 patients vs 120 patients [60.0%]; hepatocellular: 0 patients vs 45 patients [64.6%]) (Table 3 and Figure).

**Figure.**
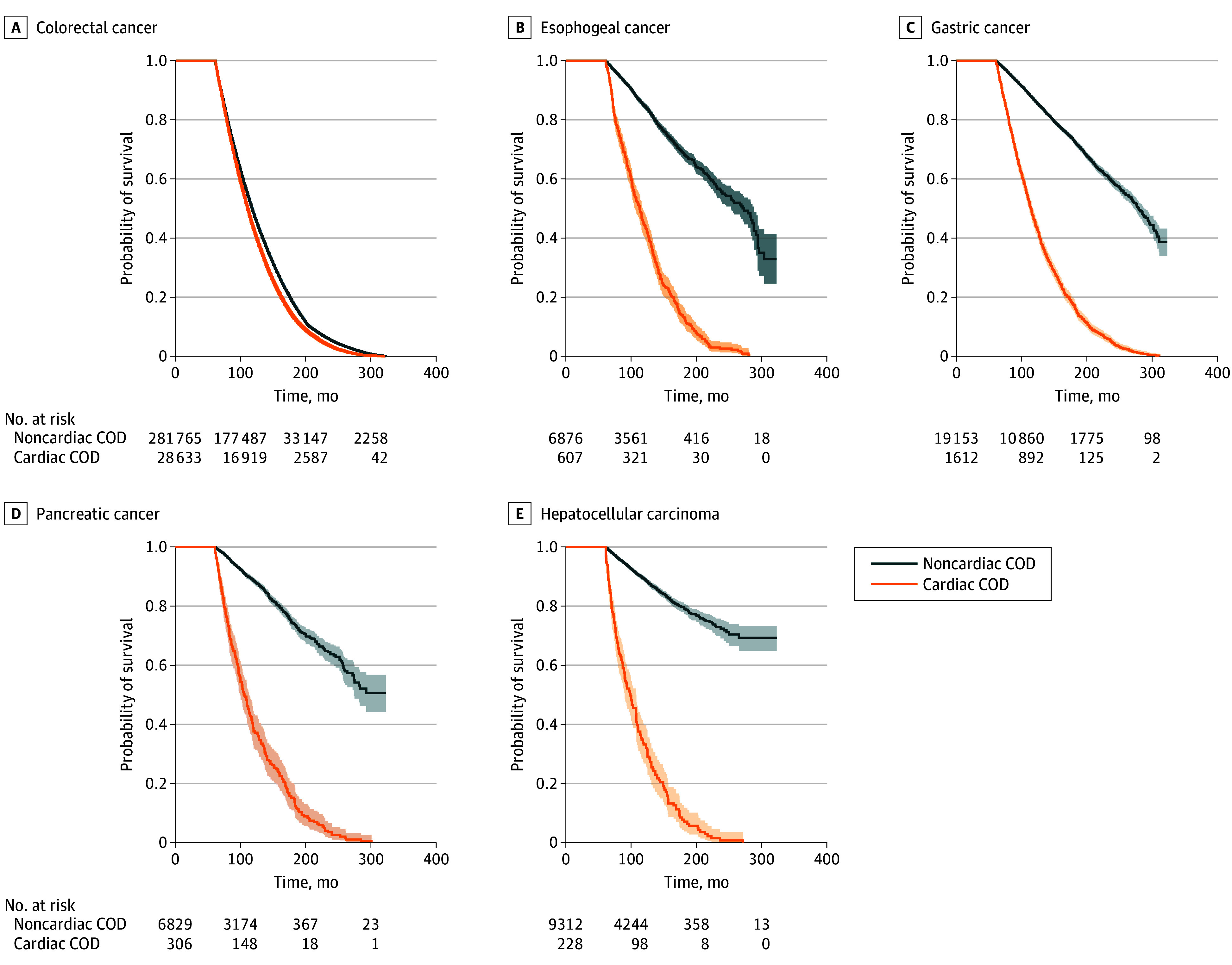
Kaplan-Meier Estimations Comparing Cardiac and Noncardiac Mortality According to Cancer Type COD indicates cause of death.

### Factors Associated With Mortality

We assessed factors associated with cardiac mortality using multivariable Cox proportion analysis. In patients with CRC, women were less likely to experience mortality (hazard ratio [HR, 0.68 [95% CI, 0.66-0.70]; *P* < .001), as were Asian or Pacific Islander individuals (HR, 0.64 [95% CI, 0.59-0.69]; *P* < .001), and patients treated with combined chemotherapy and surgery (HR, 0.68 [95% CI, 0.56-0.82]; *P* = .01). Patients with CRC were more likely to experience mortality with increasing age (HR per 1-y increase, 1.12 [95% CI, 1.12-1.12]; *P* < .001). American Indian or Alaska Native individuals with CRC were also more likely to experience cardiac mortality (HR, 1.17 [95% CI, 1.10-1.24]; *P* < .001).

For patients diagnosed with esophageal cancer, Asian or Pacific Islander individuals were 55% less likely to experience cardiac mortality (HR, 0.45 [95% CI, 0.22-0.91]; *P* = .03), while increasing age was associated with higher cardiac mortality (HR per 1-y increase, 1.08 [95% CI, 1.07-1.10]; *P* < .001). In patients with gastric cancer, women were less likely to experience mortality (HR, 0.73 [95% CI, 0.63-0.85]; *P* < .001), as well as Asian or Pacific Islander individuals (HR, 0.76 [95% CI, 0.63-0.91]; *P* < .001). Patients with gastric cancer were more likely to experience cardiac mortality with increasing age (HR per 1-y increase, 1.11 [95% CI, 1.10-1.12]; *P* < .001) if they were treated with radiation therapy alone (HR, 2.30 [95% CI, 1.03-5.12]; *P* = .04) and if they were treated with combined chemotherapy and surgery (HR, 2.14 [95% CI, 1.23-3.72]; *P* = .01).

For patients with pancreatic cancer, increasing age was associated with increased cardiac mortality (HR per 1-y increase, 1.10 [95% CI, 1.08-1.12]; *P* < .001). Additionally, in patients with hepatocellular carcinoma, women were less likely to experience mortality (HR, 0.41 [95% CI, 0.25-0.68]; *P* < .001), as were Asian or Pacific Islander individuals (HR, 0.36 [95% CI, 0.20-0.65]; *P* < .001) (Table 4).

**Table 4.** Cox Regression Analysis of Factors Associated With Cardiac Mortality Among Different Cancer Types

Variable	Colorectal cancer		Esophageal cancer		Gastric cancer		Pancreatic cancer		Hepatocellular cancer		
	HR (95% CI)	*P* value	HR (95% CI)	*P* value	HR (95% CI)	*P* value	HR (95% CI)	*P* value	HR (95% CI)	*P* value
**Characteristic**										
Sex										
Men	1 [Reference]	NA	1 [Reference]	NA	1 [Reference]	NA	1 [Reference]	NA	1 [Reference]	NA
Women	0.68 (0.66-0.70)	<.001	0.82 (0.64-1.06)	.12	0.73 (0.63-0.85)	<.001	0.85 (0.58-1.25)	.41	0.41 (0.25-0.68)	<.001
Age, per 1-y increase	1.12 (1.12-1.12)	<.001	1.08 (1.07-1.10)	<.001	1.11 (1.10-1.12)	<.001	1.10 (1.08-1.12)	<.001	1.08 (1.06-1.10)	<.001
Race										
White	1 [Reference]	NA	1 [Reference]	NA	1 [Reference]	NA	1 [Reference]	NA	1 [Reference]	NA
Black	0.89 (0.67-1.17)	.39	0.64 (0.09-4.56)	.66	1.74 (0.78-3.91)	.18	4.65 (0.63-34.42)	.13	0.72 (0.10-5.21)	.75
Asian or Pacific Islander	0.64 (0.59-0.69)	<.001	0.45 (0.22-0.91)	.03	0.76 (0.63-0.91)	.00	0.35 (0.11-1.10)	.07	0.36 (0.20-0.65)	.00
American Indian or Alaska Native	1.17 (1.10-1.24)	<.001	1.29 (0.89-1.87)	.17	1.23 (0.99-1.54)	.06	1.10 (0.51-2.38)	.81	1.44 (0.77-2.73)	.26
Tumor grade										
Differentiated										
Well I	1 [Reference]	NA	1 [Reference]	NA	1 [Reference]	NA	1 [Reference]	NA	1 [Reference]	NA
Moderately II	1.04 (0.99-1.10)	.10	0.92 (0.64-1.32)	.63	0.91 (0.72-1.16)	.46	1.18 (0.73-1.90)	.50	1.06 (0.71-1.56)	.79
Poorly III	1.05 (0.98-1.11)	.15	1.02 (0.71-1.47)	.93	1.06 (0.83-1.34)	.66	1.35 (0.75-2.41)	.32	0.61 (0.31-1.22)	.17
Undifferentiated, aplastic, IV	1.15 (0.96-1.36)	.12	1.39 (0.71-2.74)	.34	0.98 (0.57-1.67)	.93	1.29 (0.38-4.32)	.68	NA	NA
SEER summary stage										
Localized	1 [Reference]	NA	1 [Reference]	NA	1 [Reference]	NA	1 [Reference]	NA	1 [Reference]	NA
Regional	1.03 (0.99-1.06)	.14	0.81 (0.62-1.06)	.12	0.89 (0.74-1.06)	.18	1.08 (0.70-1.67)	.74	0.98 (0.57-1.67)	.93
Distant	1.08 (0.96-1.22)	.22	1.04 (0.67-1.62)	.86	0.66 (0.40-1.08)	.10	0.61 (0.23-1.62)	.32	1.41 (0.34-5.91)	.64
TNM stage (T)										
T1	1 [Reference]	NA	1 [Reference]	NA	1 [Reference]	NA	1 [Reference]	NA	1 [Reference]	NA
T2	NA	NA	0.94 (0.33-2.68)	.91	NA	NA	NA	NA	NA	NA
T3	NA	NA	NA	NA	NA	NA	NA	NA	NA	NA
T4	NA	NA	1.47 (0.50-4.32)	.48	NA	NA	0.51 (0.10-2.49)	.40	0.46 (0.05-3.97)	.48
TNM stage (N)										
N0	1 [Reference]	NA	1 [Reference]	NA	1 [Reference]	NA	1 [Reference]	NA	1 [Reference]	NA
N1	0.01 (0-1.00)	.68	1.39 (0.60-3.24)	.45	NA	NA	1.74 (0.41-7.46)	.46	NA	NA
≥N2	1.17 (1.03-1.34)	.02	1.32 (0.64-2.73)	.46	1.12 (0.43-2.91)	.82	NA	NA	1.53 (0.60-3.90)	.37
**Cancer treatment**												
Chemotherapy only										
Did not receive	1 [Reference]	NA	1 [Reference]	NA	1 [Reference]	NA	1 [Reference]	NA	1 [Reference]	NA
Received	0.64 (0.31-1.31)	.22	0.65 (0.39-1.08)	.10	0.93 (0.50-1.75)	.83	1.50 (0.56-4.05)	.42	0.61 (0.24-1.59)	.31
Radiation only										
Did not receive	1 [Reference]	NA	1 [Reference]	NA	1 [Reference]	NA	1 [Reference]	NA	1 [Reference]	NA
Received	1.28 (0.69-2.38)	.44	1.27 (0.78-2.06)	.34	2.30 (1.03-5.12)	.04	0.72 (0.08-6.32)	.77	1.24 (0.16-9.79)	.84
Surgery only										
Did not receive	1 [Reference]	NA	1 [Reference]	NA	1 [Reference]	NA	1 [Reference]	NA	1 [Reference]	NA
Received	0.91 (0.76-1.10)	.32	0.65 (0.27-1.57)	.34	0.55 (0.07-4.02)	.55	1.25 (0.41-3.81)	.70	0.64 (0.34-1.22)	.18
Combined chemotherapy and radiation										
Did not receive	1 [Reference]	NA	1 [Reference]	NA	1 [Reference]	NA	1 [Reference]	NA	1 [Reference]	NA
Received	0.98 (0.71-1.34)	.88	1.12 (0.38-3.30)	.84	0.97 (0.63-1.50)	.89	1.50 (0.33-6.73)	.60	1.70 (0.21-13.76)	.62
Combined chemotherapy and surgery										
Did not receive	1 [Reference]	NA	1 [Reference]	NA	1 [Reference]	NA	1 [Reference]	NA	1 [Reference]	NA
Received	0.68 (0.56-0.82)	.01	0.96 (0.20-4.53)	.96	2.14 (1.23-3.72)	.01	0.66 (0.03-13.64)	.79	0.47 (0.21-1.05)	.07
Combined surgery and radiation										
Did not receive	1 [Reference]	NA	1 [Reference]	NA	1 [Reference]	NA	1 [Reference]	NA	1 [Reference]	NA
Received	0.87 (0.68-1.11)	.26	NA	NA	0.71 (0.41-1.24)	.23	NA	NA	NA	NA
Combined chemotherapy, radiation, and surgery										
Did not receive	1 [Reference]	NA	1 [Reference]	NA	1 [Reference]	NA	1 [Reference]	NA	1 [Reference]	NA
Received	1.29 (0.80-2.09)	.30	NA	NA	0.90 (0.45-1.81)	.77	NA	NA	NA	NA

Abbreviations: HR, hazard ratio; NA, not applicable; SEER, Surveillance, Epidemiology, and End Results.

## Discussion

This cohort study assessed cardiac and noncardiac mortality in patients who survived more than 5 years with major GI cancers. We found that cardiac-specific mortality was higher than other causes of death in patients with colorectal esophageal, gastric, pancreatic, and hepatocellular cancers. Men were more likely to experience cardiac death compared with women. Compared with White individuals, American Indian or Alaska Native individuals were most likely to experience cardiac death compared with individuals of other races. These findings are supported by data that show American Indian or Alaska Native individuals are more likely to have higher baseline cardiovascular disease.^12,13^ Our study also found that increasing age was associated with a higher likelihood of experiencing cardiac mortality. To our knowledge, this study is the first to assess cardiac mortality in patients with major GI cancers (colorectal, esophageal, gastric, pancreatic, and hepatocellular cancer) using a large sample size.

Cardiogastroenterology is a rapidly developing field that concentrates on detecting, monitoring, and treating cardiac disease in patients with GI pathological conditions. Attention is directed toward the burden of comorbidities, improved quality of life for patients with cancer, and the effect of therapeutic agents on the induction and progression of cardiovascular disease.^14,15^ Overall, this multidisciplinary field aims to improve long-term outcomes and overall quality of life.^16^

Prior studies have shown that cancer therapy may have bimodal outcomes associated with cardiac health.^17,18^ Within the first year, typically after aggressive treatment, patients may experience pericarditis, cardiomyopathies, interruptions in the conduction pathways, and valvular dysfunction. Additionally, decades later, these same pathological conditions emerge, with the addition of coronary artery disease, all of which are associated with cardiac mortality.^17,18^

We found that cardiac survival was reduced after chemotherapy and radiation for all 5 GI cancer types. The 5 major cancers described in this study are commonly treated with antimetabolites, alkylating agents, and microtubule inhibitors. Cardiotoxic effects from fluoropyrimidines antimetabolites, including 5-fluorouracil and capecitabine, classically present with coronary spasm and ischemic heart disease.^13,19^ Alkylating agents, including cisplatin, oxaliplatin, carboplatin, and gemcitabine, have been associated with an increased risk of venous thromboembolic disease, electrical conduction disturbances, and cardiomyocyte damage from reactive oxygen species.^13,20,21,22^ Paclitaxel and docetaxel, 2 commonly used microtubule inhibitors, have been associated with arrhythmias and pericardial effusions and may potentiate the effects of anthracyclines.^13,23,24^

Hepatocellular carcinoma has been traditionally treated with sorafenib and regorafenib.^25^ However, these agents have well-established cardiotoxic effects.^26,27,28,29^ Animal studies have shown that sorafenib may result in cardiomyocyte necrosis.^30^ Furthermore, patients with pancreatic cancer are generally treated with gemcitabine and abraxane as first-line treatment for metastatic disease.^31^ However, a review of studies by Martín et al^32^ reported that patients treated with this regimen had high risk for venous thromboembolic events. Therefore, the risk of cardiac mortality in these patients may associated with the risk of thrombosis. Furthermore, a study by Khorana et al^33^ reported that in high-risk ambulatory patients with cancer (including approximately one-third of study cohort with pancreatic cancer), treatment with rivaroxaban did not significantly lower VTE incidence or death due to venous thromboembolism. Our findings further support that patients with cancer are at high risk for cardiac morbidity.

Radiation-induced heart disease is a potential sequela of oncologic treatment. Our study found that patients with esophageal cancer who received radiation had the lowest cardiac-specific survival rate (17% at 15 years). The close proximity of the esophagus with respect to the heart places the heart at risk for incidental radiation during the treatment of esophageal cancer.^34^ Clinical studies of cancer types, including breast cancer, lung cancer, and Hodgkin lymphoma, have demonstrated a consistent association between thoracic radiation and adverse cardiovascular outcomes.^35,36,37^

The effects of radiation are typically seen in a bimodal fashion. These effects are related to the radiation dose, distance from the treatment site, patient age, concomitant adjuvant therapy, and concurrent metabolic risk factors.^38^ The early manifestations of radiation-induced heart disease include pericardial effusions and pericardial inflammation; later symptoms manifest as restrictive cardiac diseases, arrhythmias, and heart failure.^38^ Radiation has been shown to increase cardiac mortality risk, especially when doses exceed 10 Gy.^39,40^ Improved therapeutic delivery techniques, such as intensity-modulated radiation therapy, are associated with a lower incidence of cardiac adverse effects; however, the burden has not been eliminated.^41,42,43,44,45^

### Limitations

This study had some limitations, One limitation was the genericity of coding for cardiac mortality in the SEER database, which death certificate irregularities and cause-of-death bias may also impact. Furthermore, without identifiable treatment regimens and comorbidities, analysis is limited by confounding factors. Demographic characteristics and access to care may also impact results. Additionally, a notable proportion of patients had missing T stage data; however, we also used SEER summary staging for assessing cancer growth.

## Conclusions

This cohort study found that patients with major GI tumors had high risk for cardiac mortality. Further research is needed to discern the mechanisms by which chemotherapy and radiation may exacerbate cardiac disease and methods for reducing risk.
